# Pneumothorax Caused by an Isolated Midshaft Clavicle Fracture

**DOI:** 10.1155/2016/2409894

**Published:** 2016-04-11

**Authors:** Najla Feriani, Hassen Ben Ghezala, Salah Snouda

**Affiliations:** Faculty of Medicine of Tunis, Tunis El-Manar University, Regional Hospital of Zaghouan, Street of Republic, 1100 Zaghouan, Tunisia

## Abstract

Patients with isolated clavicle fractures are frequent in the emergency department. However, unusual clavicle fractures complications, such as pneumothorax, are rare. Previous reports indicated that all pneumothorax cases were treated via performing thoracostomy. Conservatively, the treatment of the clavicle fracture, like in our case, was successful. Despite the fact that isolated clavicle fractures rarely cause complications and generally heal with immobilization, serious complications may occur requiring urgent treatment. It has been proven that physical examinations, with particular attention to the neurovascular and chest examinations, and radiographs of the clavicle are necessary to prevent overlooking these potentially dangerous complications.

## 1. Introduction

Fractures of the clavicle are relatively common, representing 2.6% to 4% of fractures among adults [1–3]. The majority of these fractures occur in the middle third of the shaft. They are the result of low-energy mechanisms such as a fall onto the shoulder [2–5]. Pneumothorax, as a consequence of a clavicle fracture, is a rare but potentially lethal complication [6]. We report in this work an exceptional case of isolated clavicle fracture causing a pneumothorax, which required the insertion of a chest drain.

## 2. Case Report

A 30-year-old male was admitted to the emergency department of our hospital, after a sideslip of his car. During the medical examination, he complained of a pain in the left shoulder. There was no relevant medical history. The patient symptoms and behavior revealed no clinical distress. There were no neurovascular deficits in the right upper limb. The lung breath sounds and percussion notes were normal on the right shoulder and low on the left one.

The patient denied any chest discomfort, dyspnea, or hemoptysis. He had moderate central and peripheral cyanosis. The respiratory rate was 24 breaths per minute. A pulse oximetry monitor showed an oxygen saturation level of 92 percent. The arterial blood pressure was 130/70 mmHg and the pulse was 95 bpm. Any further physical examination was unremarkable. The Glasgow coma scale was 15 with a normal neurological examination.

The initial laboratory data included serum hemoglobin of 125 g/L and hematocrit of 43%. The white blood cell count was normal at 9000 cells per microliter with a normal platelet count of 326 billion platelets per liter of blood. Laboratory studies included normal prothrombin time, activated partial thromboplastin time, normal platelet functional analysis, and negative disseminated intravascular coagulation screen. Serum electrolyte levels and renal function were normal. Arterial blood gas analysis showed a mild hypoxemia with a PaO_2_ of 78 mmHg and a SaO_2_ greater than 96%. The patient had a normal acid-base balance; the pH was 7.42 with a serum bicarbonate level at 22 mEq/L. The blood gas carbon dioxide level was normal at 36 mmHg.

**Figure 1:**
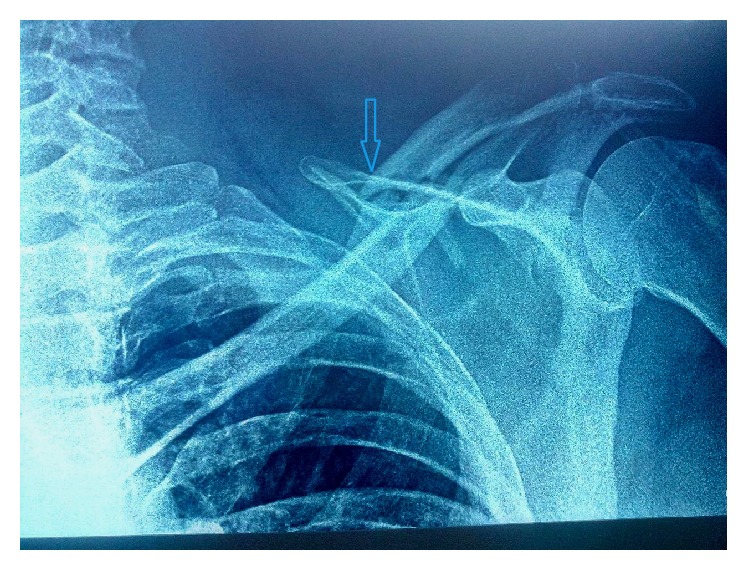
Clavicle radiography showing the clavicle fracture.

**Figure 2:**
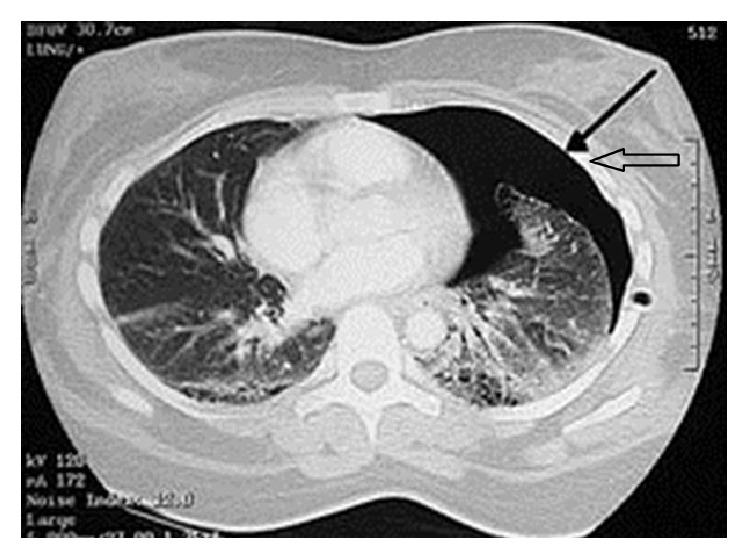
Chest CT scan showing left-sided pneumothorax.

Radiographs of the clavicle (Figure 1) showed a displaced comminuted midshaft fracture. There were no rib fractures. The patient was admitted to the hospital. A computed tomographic scan revealed a left-sided pneumothorax (Figure 2).

**Figure 3:**
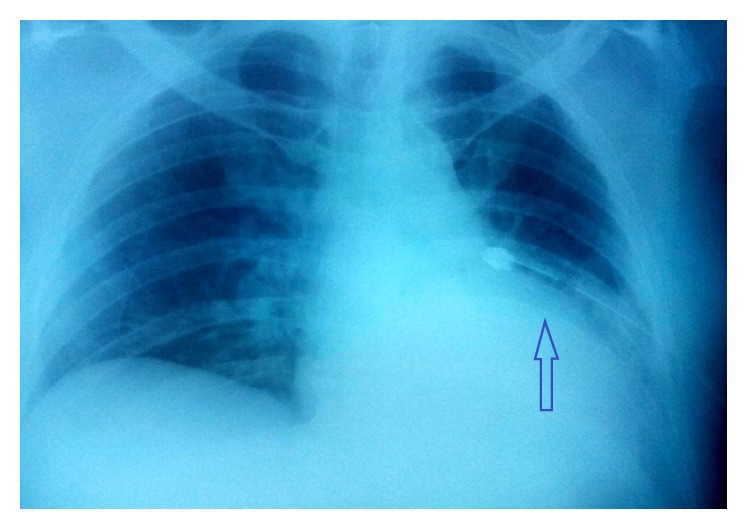
Chest X-ray after insertion of chest tube.

The pneumothorax was treated by the insertion of a chest drain under local anesthesia (Figure 3). In this last chest X-ray a small hemothorax appeared. This could be due to the insertion of the chest drain after we made the diagnosis of pneumothorax. The chest drain was removed five days after the injury and the patient was discharged home in a stable condition with the right arm resting in a sling. Two months after the accident, the chest radiograph showed that the clavicle fracture had consolidated in a correct position.

## 3. Discussion

Our case is rare. It reports an exceptional case of clavicle fracture complicated by pneumothorax without any lung posttraumatic lesions.

Clavicle fractures are relatively common. There is a significant difference in age-specific incidence of fractures of different anatomic parts of the clavicle. Allman Group 1, middle third fractures are most common in children and young adults; Allman Group 2, third lateral fractures are most frequent among the middle-aged; and Group 3 fractures, affecting the medial third, are most common among the elderly [2, 4, 7].

Nonunion and malunion, with clinical deformity, are the most common problems that can be caused by this injury. Anatomically, the apex of the lung lies behind and above the medial one-third of the clavicle, with the anterior scalene muscle, brachial plexus, and subclavian vessel interferences. However, the incidence of complications associated with isolated clavicle fractures, including vascular, brachial plexus, and pneumothorax, is low (1–3%) [8].

Clavicular fractures most frequently result from a direct injury as a fall onto the shoulder [6]. Like in our case presentation, it is interesting to note that three of five reported cases in the literature of pneumothorax complicating clavicular fractures were caused by direct injury of low velocity [9–11].

A careful history and physical examination with particular attention to the neurovascular and chest examination are vital [9, 10, 12, 13]. In trauma, routine chest radiographs are part of the initial imaging series; however, patients presenting with isolated injuries may not receive a standard chest film. Making a chest radiograph part of the standard imaging series in these patients may be necessary and warrants further investigation [11].

Most fractures of the clavicle can be treated conservatively with good results. Fractures sustained by adults are usually harder to treat than those of children. The bones displace more easily and take longer to heal. Nevertheless, operative treatment is only occasionally indicated, in cases with a risk of perforation of the skin, neurovascular complications, and pseudarthrosis. Most complications occur with operative treatment and include nonunion and infection. Neurovascular complications are uncommon but not exceptional [6]. Usually, clavicle fractures heal uneventfully and are rarely complicated by significant morbidity [14].

In our case, the patient did not have major respiratory complaints and radiographs of the clavicle showed a displaced midshaft fracture of the left clavicle. A significant pneumothorax requiring emergency treatment by chest drain insertion was revealed only by chest CT scan. The initial chest X-ray did not find it and after several weeks, the patient recovered totally.

## 4. Conclusion

Patients who have sustained a clavicle fracture should be evaluated for concomitant chest, neurologic, and vascular injury. In addition to physical examination, chest radiography should be considered in the evaluation of patients with displaced clavicle fractures. In fact, as shown in our case report, pneumothorax should be considered as a potential complication and must be excluded by appropriate clinical examination and radiographs.

## References

[1] Lohse G. R., Lee D. H. (2013). Clavicle fracture with intrathoracic displacement. *Orthopedics*.

[2] Nordqvist A., Petersson C. (1994). The incidence of fractures of the clavicle. *Clinical Orthopaedics and Related Research*.

[3] Postacchini F., Gumina S., De Santis P., Albo F. (2002). Epidemiology of clavicle fractures. *Journal of Shoulder and Elbow Surgery*.

[4] Robinson C. M. (1998). Fractures of the clavicle in the adult. Epidemiology and classification. *The Journal of Bone and Joint Surgery—British Volume*.

[5] Stanley D., Trowbridge E. A., Norris S. H. (1988). The mechanism of clavicular fracture. A clinical and biomechanical analysis. *The Journal of Bone & Joint Surgery—British Volume*.

[6] Steenvoorde P., Van Lieshout A. P. W., Oskam J. (2005). Conservative treatment of a closed fracture of the clavicle complicated by pneumothorax: a case report. *Acta Orthopaedica Belgica*.

[7] Nowak J., Mallmin H., Larsson S. (2000). The aetiology and epidemiology of clavicular fractures: a prospective study during a two-year period in Uppsala, Sweden. *Injury*.

[8] Rowe C. R. (1968). An atlas of anatomy and treatment of midclavicular fractures. *Clinical Orthopaedics and Related Research*.

[9] Dath R., Nashi M., Sharma Y., Muddu B. N. (2004). Pneumothorax complicating isolated clavicle fracture. *Emergency Medicine Journal*.

[10] Yates D. W. (1976). Complications of fractures of the clavicle. *Injury*.

[11] Tjoumakaris F. P., Matzon J. L., Williams G. R. (2011). Clavicle fracture with thoracic penetration and hemopneumothorax but without neurovascular compromise. *Orthopedics*.

[12] Williams R. J. (1995). Significant pneumothorax complicating a fractured clavicle. *Journal of Accident & Emergency Medicine*.

[13] Meeks R. J., Riebel G. D. (1991). Isolated clavicle fracture with associated pneumothorax: a case report. *American Journal of Emergency Medicine*.

[14] Mouzopoulos G., Morakis E., Stamatakos M., Tzurbakis M. (2009). Complications associated with clavicular fracture. *Orthopaedic Nursing*.

